# Collecting Social Determinants of Health in a Children’s Oncology Group Trial for High-Risk Neuroblastoma

**DOI:** 10.1001/jamanetworkopen.2026.0419

**Published:** 2026-03-02

**Authors:** Emily Jones, Arlene Naranjo, Lena E. Winestone, Puja J Umaretiya, Rahela Aziz-Bose, Colleen A. Kelly, Haley Newman, Daniel J. Zheng, Emily Greengard, Rochelle Bagatell, Steven G. DuBois, Kira Bona

**Affiliations:** 1 Dana-Farber Boston Children’s Cancer and Blood Disorders Center, Boston, Massachusetts; 2 Children’s Oncology Group Statistics and Data Center, University of Florida, Gainesville; 3 UCSF Benioff Children’s Hospital, San Francisco, California; 4 Department of Pediatrics, University of Texas Southwestern Medical Center, Dallas; 5 Division of Oncology, Department of Pediatrics, Children’s Hospital of Philadelphia and Perelman School of Medicine, Philadelphia, Pennsylvania; 6 Children’s Minnesota, Minneapolis

## Abstract

This cohort study examines the feasibility and acceptability of collecting parent-reported social determinants of health (SDOH) data in a Children’s Oncology Group (COG) trial.

## Introduction

Children with cancer exposed to adverse social determinants of health (SDOH), including poverty, experience outcome disparities. Medicaid insurance, a proxy for low-income, is independently associated with inferior event-free and overall survival—despite receipt of uniform planned treatment on National Clinical Trials Network (NCTN) Children’s Oncology Group (COG) trials.^1,2^ Sociodemographic data collected in NCTN trials—race, ethnicity, and insurance—serve as proxies for exposure to adverse SDOH but are prone to misclassification, and can neither identify mechanisms underlying disparities nor provide modifiable intervention targets. Household material hardship (HMH)—unmet basic needs including food, housing, utility, or transportation insecurity—is a parent-reported SDOH exposure associated with inferior general pediatric health outcomes^3^ and modifiable with intervention at the policy, system, and household levels.^4^ We evaluated the feasibility and acceptability of parent-reported SDOH data collection in an international COG NCTN trial.

## Methods

This prospective cohort study was an embedded aim of the Testing the Addition of 131I-MIBG or Lorlatinib to Intensive Therapy in People with High-Risk Neuroblastoma trial (NCT03126916), a groupwide, randomized phase 3 trial for de novo high-risk neuroblastoma (2018-2023). Beginning in 2019, patients younger than 18 years at US or Canadian sites were eligible to opt in at initial trial consent to a single time point SDOH survey to evaluate HMH and outcomes. Parents or guardians completed a 27-item, paper survey during the first chemotherapy cycle at treating sites (eMethods in Supplement 1). Surveys could be administered in any language with an interpreter and were available in English, Spanish, and French.^5^ Neither sites nor participants received remuneration. Responses were collected as research data, thus were not evaluated in real-time nor shared with treating teams. The trial was approved by the NCI Pediatric central and local IRBs. Written informed consent and assent were obtained at trial enrollment. This report follows the STROBE guideline.

Feasibility was evaluated by the proportion of eligible participants who opted in and the proportion of completed surveys. Acceptability was characterized by HMH data completeness—the prespecified primary SDOH exposure for trial analyses. Characteristics of participants and nonparticipants were compared; continuous variables were compared with the Wilcoxon rank sum test and categorical variables with the χ^2^ test, or if small cell sizes, Fisher exact test. A 2-sided *P* < .05 was considered significant. Analyses were performed in SAS version 9.4 (SAS Institute).

## Results

Among 519 eligible participants, 459 (88.4%) opted in across 114 sites. Participant and nonparticipant characteristics did not differ (Table 1). Among opt-in participants, 417 (90.8%) completed the survey in 8 languages, a median (range) of 12 (0-86) days postconsent. Missed surveys were due to participant decline (7 participants [1.5%]), COVID-19 restrictions (5 participants [1.1%]), scheduling (6 participants [1.3%]), off protocol therapy or study (15 participants [3.3%]), and unknown (9 participants [2.0%]). HMH data were 98.6% complete, with 30.5% of the cohort (127 participants) reporting HMH-exposure (Table 2).

**Table 1.** SDOH Correlative Study Participant and Nonparticipant Demographics

Patient characteristics	Participants elgigible for SDOH correlative study, No. (%) (N = 519)^a^		*P* value^b^
	Participants (n = 417)	Nonparticipants (n = 102)	
Age at diagnosis, median (range), y	3.3 (0.3-17.4)	3.1 (1.1-12.2)	.55
Sex			
Female	186 (44.6)	41 (40.6)	.47
Male	231 (55.4)	60 (59.4)	
Unknown	0	1 (<0.0)	
Race^c^			
American Indian or Alaska Native	4 (1.1)	1 (1.1)	.80
Asian	24 (6.7)	3 (3.4)	
Black or African American	55 (15.3)	14 (16.1)	
Native Hawaiian or Other Pacific Islander	1 (0.3)	0	
White	268 (74.4)	66 (75.9)	
>1 Race	8 (2.2)	3 (3.4)	
Missing or not known, No.	57	15	
Ethnicity^c^			
Hispanic or Latino/a	74 (19.8)	11 (12.4)	.10
Not Hispanic or Latino/a	300 (80.2)	78 (87.6)	
Missing or not known, No.	43 (10.3)	13 (12.7)	
Insurance^d^			
Public	141 (36.2)	35 (36.8)	.91
Private	248 (63.8)	60 (63.2)	
Missing or not known, No.	28	7	

Abbreviation: SDOH, social determinants of health.

^a^ Those included in the participant category include 417 eligible participants who opted in to SDOH correlative study and completed the Household Survey. Those included in the nonparticipant category include 60 eligible participants who declined SDOH correlative study participation, and 42 eligible participants who opted in to SDOH correlative study participation but did not complete the Household Survey. Percentages calculated excluding missing or unknown categories.

^b^ Tests exclude missing or unknown categories. Continuous variables were compared with the Wilcoxon rank sum test, categorical variables were compared with the χ^2^ test or if small cell sizes (ie, for race), Fisher exact test. *P* values are 2-sided and considered significant if <.05.

^c^ Race and ethnicity were obtained from trial case report forms collected per US Office of Management and Budget guidelines. Race and ethnicity were not collected on the Household Survey.

^d^ Public insurance was defined as US Medicaid only. Private insurance included all of the following trial-specified categories: (1) Medicaid and Medicare, (2) Medicare, (3) Medicare and private insurance, (4) military or veterans sponsored, (5) military sponsored (including Civilian Health and Medical Program of the Uniformed Services and TRICARE), (6) no means of payment (no insurance), (7) other, (8) private insurance, and (9) self-pay (no insurance). Trial-specified insurance categories did not include a separate Canadian provincial category.

**Table 2.** Characteristics of HMH Among SDOH Correlative Study Participants Who Reported HMH Exposure^a^

Characteristic	Participants, No. (%) (N = 417)
Any HMH^b^	127 (30.5)
HMH severity^c^	
Mild (1 domain)	49 (11.8)
Severe (≥2 domains)	78 (18.7)
HMH domain^b^	
Food insecurity	70 (16.8)
Housing insecurity	69 (16.5)
Utility insecurity	43 (10.3)
Transportation insecurity	32 (7.7)

Abbreviations: HMH, household material hardship; SDOH, social determinants of health.

^a^ Those included as participants include 417 eligible participants who opted in to the SDOH correlative study and completed the Household Survey.

^b^ Families were considered to have HMH if they reported at least 1 of 4 concrete needs: food insecurity, housing insecurity, utility insecurity, or transportation insecurity.

^c^ HMH is scored in ordinal categories (0-4) based on number of unmet basic needs (food, housing, utilities, and transportation) using a 5-item instrument. Scores of 1 to 4 are HMH-exposed, dichotomized as mild (1 domain) vs severe (2-4 domains).

## Discussion

This cohort study found that collecting parent-reported SDOH data in a groupwide COG NCTN clinical trial is feasible and acceptable, with an 88.4% opt-in rate, 90.8% survey completion rate, and minimal data missingness. Over 98% of participants were willing to share data on unmet basic needs for research, without the possibility of benefit for themselves, and 1 in 3 families reported HMH.

Despite planned uniform treatment on cooperative group trials, children exposed to proxied SDOH are more likely to relapse and die.^1^ Collecting modifiable SDOH data as an expected component of NCTN trials is a pivotal next step to strengthen external validity (generalizability of toxic effects and response), improve internal validity (incorporation of SDOH alongside genomic and other biologic prognosticators), and identify populations who warrant targeted interventions to improve outcomes. Feasibility was evaluated within a disease-specific US and Canadian population, and a limited SDOH dataset was captured; thus, results may not generalize to broader social risk assessments or other settings. Nonetheless, groupwide feasibility supports extrapolation across NCTN trials. Integrating embedded, family-reported SDOH data collection into future trials is essential to identify mechanisms underlying treatment failure and to inform health equity interventions to improve outcomes.

## Notes

Supplement 1. eMethods. Household Survey

Supplement 2. Data Sharing Statement
