# Supplementary material for: Collecting Social Determinants of Health in a Children’s Oncology Group Trial for High-Risk Neuroblastoma
Source: JAMA Netw Open. 2026 Mar 2;9(3):e260419. doi: 10.1001/jamanetworkopen.2026.0419 (PMC12954535; doi:10.1001/jamanetworkopen.2026.0419)
Supplement: Supplement 1. — eMethods. Household Survey [file jamanetwopen-e260419-s001.pdf]

Supplemental Online Content

Jones E, Naranjo A, Winestone LE, et al. Feasibility of collecting social determinants of health in a Children's Oncology Group trial. *JAMA Netw. Open*. 2026;9(3):e260419. doi:10.1001/jamanetworkopen.2026.0419

eMethods. Household Survey

This supplemental material has been provided by the authors to give readers additional information about their work.

© 2026 Jones E et al. *JAMA Network Open*.

eMethods. Household Survey

Parents/guardians of opt-in study participants completed a 27-item, single-timepoint “Household Survey” during the first Induction chemotherapy cycle. Paper/pencil surveys were available in US (English and Spanish) and Canadian (English and French) versions; surveys could be administered in any language with an interpreter. Survey domains included language, health literacy, marital status, education, household material hardship (HMH), household income, employment status, and social support. Characteristics of participants (those who opted in and completed the Household Survey) vs nonparticipants (those who declined participation or opted-in but did not complete the survey) were compared.

Baseline Survey

Nearly 1 in 3 families experience problems with money or financial stress during their child's cancer treatment. This may include lost income from work, difficulty paying the rent or mortgage, difficulty keeping the electricity or heat on or putting food on the table. Financial stresses or problems with money can affect both adults and children in many ways. Study doctors are hoping to learn about how common these stresses are at the beginning of neuroblastoma therapy, and to make sure that financial stress does not impact a child's experience during treatment. Information from families like yours will help us achieve this goal.

This survey has multiple-choice questions and will take about 5 minutes to complete. The survey includes questions about you and your family, housing, home utilities (heaVelectricity), food, finances and family supports. We understand that some of these questions may feel personal and difficult to answer. You may skip any questions and even stop completing the survey at any time. All the information you provide will help us to better understand the range of family experiences at the start of neuroblastoma treatment.

All of your survey answers will be confidential. Your answers will <u>not</u> be shared with your treatment team. Survey data will <u>not</u> be placed in your child's medical record.

Before we analyze the survey data for research they will be de-identified, which means that no information that would permit identification of your family or child will be shared.

To be completed by administering research staff.

Today's date (month/day/year): _____/______/ ____ _

Name of staff member administering survey (Last, First): ________________________

Site at which survey administered: ________________________

Survey administered:

a. English
b. Spanish
c. French

Interpreter used:

© 2026 Jones E et al. *JAMA Network Open*.

a. Yes (specify language): _______________
b. No

© 2026 Jones E et al. *JAMA Network Open*.

A. Demographics

The following questions ask you a bit about yourself and your family.

1. What is your relationship to the patient?
   a. Mother
   b. Father
   c. Other Legal Guardian (please specify): ______________________________

2. What is the primary language you speak at home?
   a. English
   b. Spanish
   c. French
   d. Other, please specify: ____________________

3. How confident are you filling out medical forms by yourself?
   a. Not at all
   b. A little bit
   c. Somewhat
   d. Quite a bit
   e. Extremely

4. Please describe your marital status:
   a. Single (living alone)
   b. Married or living together with partner
   c. Widowed
   d. Separated or divorced
   e. Other: ________

5. What is the highest level of education you received? *Please choose the best response:*
   a. Grade school/elementary school (Grades 1-8)
   b. Started high school but didn't finish (Grades 9-12)
   c. High school diploma or GED
   d. Trade/technical/vocational training after high school
   e. Some college
   f. College graduate
   g. Postgraduate (Master's or Doctorate)

6. If your child has another caregiver/parent (specify relationship: ____________), what is the highest level of education he/she received? *Please choose the best response:*
   a. Not applicable
   b. Grade school/elementary school (Grades 1-8)
   c. Started High School but didn't finish (Grades 9-12)
   d. High school diploma or GED
   e. Trade/technical/vocational training after High School
   f. Some college
   g. College graduate
   h. Postgraduate (Master's or Doctorate)

About your child:

7. In general, how would you rate your child's health before his/her neuroblastoma diagnosis?
   a. Excellent
   b. Very good
   c. Good
   d. Fair
   e. Poor

© 2026 Jones E et al. *JAMA Network Open*.

B. Housing and Transportation

*This part of the survey asks about your child's housing **before his/her neuroblastoma diagnosis.** If you have moved since your child was diagnosed with cancer, please answer these questions **about the housing in which your child lived before his/her diagnosis.***

1. Please tell me what type of housing you and your child live in (choose the best response):
   a. Apartment
   b. House/Townhouse/Condo
   c. Mobile home/trailer
   d. Room/rented room
   e. Shelter or transitional living situation
   f. No steady place to sleep at night (e.g., temporarily staying with others, in a hotel, on the street, in a car, in a park etc)
   g. Other (please specify): ___________________

2. Before your child's diagnosis, how many people (including yourself and your child) were living in your household?
   ____________Number of people
   ____________Of these people, how many are children (ages 0-17 years old)?
   ____________Of these people, how many are adults (ages >=18 years old)?
   ____________Of the adults, how many bring income into the household?

3. In the 6 months before your child's diagnosis, was there a time when you were not able to pay the rent or mortgage on time because of financial difficulties?
   a. Yes
   b. No
   c. Not applicable (I don't pay rent or mortgage)

4. In the 6 months before your child's diagnosis, did lack of reliable transportation keep you or anyone in your family from medical appointments, meetings, work or getting things needed for daily living?
   a. Yes
   b. No
   c. Not Sure/Don't know

C. Utilities

*The following questions ask about heat and electricity in your home. Please answer these questions thinking about the 6 months **before your child was diagnosed with neuroblastoma.***

1. During the past 6 months, has the gas/electric/oil company sent you a letter <u>threatening to shut off or not delver</u> the gas/electricity/oil to the house for not paying bills?
   a. Yes
   b. No
   c. Not applicable, I don't pay for gas/electric/oil

2. During the past 6 months, has the gas/electric/oil company <u>shut off</u> electricity/gas or refused to deliver oil for not paying bills?
   a. Yes
   b. No
   c. Not applicable, I don't pay for gas/electric/oil

© 2026 Jones E et al. *JAMA Network Open*.

D. Food

The next set of questions are about the food eaten in your household in the last 6 months, and whether you were able to afford the food you need. Please answer these questions thinking about ***the 6 months before your child was diagnosed with neurob/astoma.***

I'm going to read you two statements that other people have made about their food situation. For each statement, please tell me whether the statement was often true, sometimes true, or never true for your household in the last 6 months.

1. The first statement is, "We worried whether our food would run out before we got money to buy more." Was that often, sometimes, or never true for your household in the last 6 months?
   a. Often true
   b. Sometimes true
   c. Never true
   d. Don't know or prefer not to answer

2. "The food that we bought just didn't last, and we didn't have money to get more." Was that often, sometimes, or never true for your household in the last 6 months?
   a. Often true
   b. Sometimes true
   c. Never true
   d. Don't know or prefer not to answer

E. Finances

The next set of questions are about the financial resources supporting your child. Please answer these questions thinking about your household ***before your child's neuroblastoma diagnosis.***

1. Please provide your best estimate of your tot al combined household income (i.e. the amount your family would report on your taxes). This should include income from all sources: wages, child support payments, rent from properties, social security, disability and/or veteran's benefits, unemployment benefits, workman's compensation, and so on:
   a. $ ____________
   b. Don't know or prefer not to answer

2. If your family lost all of your current sources of income (for example, your paycheck, Social Security or pension, public assistance) and had to live off of your savings, how long could you continue to live at your current address and standard of living?
   a. Less than 1 month
   b. 1-2 months
   c. 3-6 months
   d. 7-12 months
   e. More than 1 year
   f. Don't know

3. Which of the following best describes your main daily activities and/or responsibilities **before your child's diagnosis?**
   a. Working full time
   b. Working part time
   c. Unemployed or laid off
   d. Looking for work
   e. Raising children full-time or keeping house
   f. Retired
   g. Other (specify): ___________

© 2026 Jones E et al. *JAMA Network Open*.

4. If your child has another parent/guardian, which of the following best describes his/her main daily activities and/or responsibilities **before your child's diagnosis?**

a. Not applicable
b. Working full time
c. Working part time
d. Unemployed or laid off
e. Looking for work
f. Raising children full-time or keeping house
g. Retired
h. Other (specify): ___________

F. Social Support

People often look to others for companionship, assistance, or other types of support. Thinking about life before your child's diagnosis, how often was each of the following kinds of support available to you if you needed it? Choose one number from each line.

How often is someone available:	None of the time	A little of the time	Some of the time	Most of the time	All of the time
To help you if you were confined to bed?	1	2	3	4	5
To take you to the doctor if you need it?	1	2	3	4	5
To prepare your meals if you are unable to do it yourself?	1	2	3	4	5
To help with daily chores if you were sick?	1	2	3	4	5
To have a good time with?	1	2	3	4	5
To turn to for suggestions about how to deal with a personal problem?	1	2	3	4	5
Who understands your problems?	1	2	3	4	5
To love and make you feel wanted?	1	2	3	4	5

Any additional comments:

____

____

© 2026 Jones E et al. *JAMA Network Open*.
