# Supplementary material for: Collecting Social Determinants of Health in a Children’s Oncology Group Trial for High-Risk Neuroblastoma
Source: JAMA Netw Open. 2026 Mar 2;9(3):e260419. doi: 10.1001/jamanetworkopen.2026.0419 (PMC12954535; doi:10.1001/jamanetworkopen.2026.0419)
Supplement: Supplement 2. — Data Sharing Statement [file jamanetwopen-e260419-s002.pdf]

Data Sharing Statement

Jones. Feasibility of Collecting Social Determinants of Health in a Children's Oncology Group Trial. *JAMA Netw Open*. Published March 02, 2026. doi:10.1001/jamanetworkopen.2026.0419

Data
Data available: No

Additional Information
Explanation for why data not available: The Children's Oncology Group Data Sharing policy describes the release and use of COG individual subject data for use in research projects in accordance with National Clinical Trials Network (NCTN) Program and NCI Community Oncology Research Program (NCORP) Guidelines. Only data expressly released from the oversight of the relevant COG Data and Safety Monitoring Committee (DSMC) are available to be shared. Data sharing will ordinarily be considered only after the primary study manuscript is accepted for publication. For phase 3 studies, individual-level de-identified datasets that would be sufficient to reproduce results provided in a publication containing the primary study analysis can be requested from the NCTN/NCORP Data Archive at https://nctn-data-archive.nci.nih.gov/. Data are available to researchers who wish to analyze the data in secondary studies to enhance the public health benefit of the original work and agree to the terms and conditions of use. For non-phase 3 studies, data are available following the primary publication. An individual-level de-identified dataset containing the variables analyzed in the primary results paper can be expected to be available upon request. Requests for access to COG protocol research data should be sent to: datarequest@childrensoncologygroup.org. Data are available to researchers whose proposed analysis is found by COG to be feasible and of scientific merit and who agree to the terms and conditions of use. For all requests, no other study documents, including the protocol, will be made available and no end date exists for requests. In addition to above, release of data collected in a clinical trial conducted under a binding collaborative agreement between COG or the NCI Cancer Therapy Evaluation Program (CTEP) and a pharmaceutical/biotechnology company must comply with the data sharing terms of the binding collaborative/contractual agreement and must receive the proper approvals.
